# Characterization of primary normal and malignant breast cancer cell and their response to chemotherapy and immunostimulatory agents

**DOI:** 10.1186/s12885-018-4635-8

**Published:** 2018-07-09

**Authors:** Anna A. Nushtaeva, Grigory A. Stepanov, Dmitry V. Semenov, Evgeny S. Juravlev, Evgenia A. Balahonova, Alexey V. Gerasimov, Sergey V. Sidorov, Eugeniy I. Savelyev, Elena V. Kuligina, Vladimir A. Richter, Olga A. Koval

**Affiliations:** 1 Institute of Chemical Biology and Fundamental Medicine, Siberian Branch, Russian Academy of Sciences, Lavrentiev Avenue, 8, 630090 Novosibirsk, Russia; 2 Novosibirsk State University, Pirogova str., 1, 630090 Novosibirsk, Russia; 3 National Novosibirsk Regional Oncology Dispensary, Plakhotnogo str., 2, 630000 Novosibirsk, Russia; 4 Novosibirsk Municipal Budgetary Healthcare Institution “Municipal Clinical Hospital #1”, Zalessky str., 6, 630047 Novosibirsk, Russia; 5 Center of New Medical Technologies, Pirogova, str., 25/4, 630090 Novosibirsk, Russia

**Keywords:** Breast cancer, Primary culture, Hormone receptor, Prognostic marker, Cancer stem cells, CD44, CD24, *IFIT3*, Interferon-α, snoRNA

## Abstract

**Background:**

The phenomenon of chemotherapy-resistant cancers has necessitated the development of new therapeutics as well as the identification of specific prognostic markers to predict the response to novel drugs. Primary cancer cells provide a model to study the multiplicity of tumourigenic transformation, to investigate alterations of the cellular response to various molecular stimuli, and to test therapeutics for cancer treatment.

**Methods:**

Here, we developed primary cultures of human breast tissue – normal cells (BN1), cancer cells (BC5), and cells from a chemotherapy-treated tumour (BrCCh1) to compare their response to conventional chemotherapeutics and to innate immunity stimulators with that of the immortalized breast cells MCF7, MDA-MB-231, and MCF10A. Expression of the progesterone receptor (PGR), oestrogen receptor (ER) α and β, human epidermal growth factor receptor (HER) 2 and 3 and aromatase CYP19, as well as expression of interferon-induced protein with tetratricopeptide repeats 3 (*IFIT3*) mRNA in human breast cells were characterized.

**Results:**

We revealed that BC5 carcinoma cells were PGR^low^/ERb^high^/ERa^−^/Cyp19^+^, the BrCCh1 cells that originated from the recurrent tumour were PGR^−^/ERb^+^/ERa^−^/Cyp19^+^, and normal BN cells were PGR^−^/ERb^+^/ERa^−^/Cyp19^high^. The treatment of primary culture cells with antitumour therapeutics revealed that BrCCh1 cells were doxorubicine-resistant and sensitive to cisplatin. BC5 cells exhibited low sensitivity to tamoxifen and cisplatin. The innate immunity activators interferon-α and an artificial small nucleolar RNA analogue increased expression of *IFIT3* at different levels in primary cells and in the immortalized breast cells MCF7, MDA-MB-231, and MCF10A. The relative level of activation of *IFIT3* expression was inversely correlated with the baseline level of *IFIT3* mRNA expression in breast cell lines.

**Conclusion:**

Our data demonstrated that primary cancer cells are a useful model for the development of novel cancer treatments. Our findings suggest that expression of *IFIT3* mRNA can be used as a prognostic marker of breast cancer cell sensitivity to immunostimulating therapeutics.

## Background

Breast cancer was the leader among new cancer cases in the United States in 2016 and the second highest cause of death among women [1]. Cancer development is a multi-step process where various oncogenic mutations give rise to cancer cells with different genetic defects, which can differ even within an individual tumour. This diversity is a significant obstacle in cancer treatment, so cell lines established from human tumour samples can be a beneficial tool for screening relevant drugs. For prognosis of cancer sensitivity to certain therapeutics, direct cytotoxicity assays in cancer cells can be combined with data regarding the expression of genes essential for antitumour action [2]. The significant characteristics of individual breast cancers are the expression of oestrogen, progesterone, and human epidermal growth factor receptor 2 (HER2/CD340), and the proposed type of treatment is based in part on these characteristics [3]. Among them, oestrogen receptor (ER) status is more critical for predicting the response to hormonal therapy, whereas progesterone receptor (PGR) status has remained controversial [4, 5]. Double-positive ER^+^/PGR^+^ breast cancer has shown better outcomes than single-positive tumours. HER2 expression allows for the choice of anti-HER2 targeted therapy, whereas the triple negative breast cancer (TNBC) subtype is usually more aggressive, with the worst prognosis, as targeted or hormonal therapy is not available and patients are treated with standard chemotherapy or radiotherapy [6].

A novel approach to cancer therapy arises from the existence of cancer stem cells (CSCs), which display a drug resistant phenotype. It has been reported that recurrent breast tumours are driven by a subpopulation of tumour-initiating CSCs. A subpopulation of cancer cells with a CD44^+^/CD24^−/low^ phenotype has stem/progenitor cell features that are associated with a poor outcome in patients [7, 8]. In vitro screening of drug sensitivity can help to reduce the chemotherapeutic dose and decrease the toxic effects of low-effective chemotherapeutics, including TNBC subtypes and CSC-reach cancers. This approach can decrease the immunosuppressive effects of chemotherapy and as a consequence, the immune system would recognize transformed cells in order to inhibit the growth of neoplastic tissue [9]. In such way immunosurveillance possible to make contribution against cancer.

Over the past few years, immunotherapy has become a part of a complex approach for the treatment of malignant diseases. In addition to new T-cell adoptive transfer and monoclonal antibodies, interferons (IFNs) and cytokines can produce therapeutic responses [10, 11]. IFNα is used to treat hairy-cell leukaemia, renal carcinoma, myeloma, and melanoma, whereas IFNγ has been approved for the treatment of ovarian, renal, and endometrial cancers [12]. Because of the broad spectrum of individual side effects and the toxicity that accompany IFN therapy, new molecules with immunomodulatory properties that can trigger innate immunity and initiate apoptosis of cancer cells are being studied.

RNAs have been shown to modulate various cellular responses as well as induce apoptosis of cancer cells in vitro [13, 14]. Recently, it was demonstrated that the level of expression of hepatic interferon-induced protein with tetratricopeptide repeats 3 (*IFIT3*) mRNA predicts the IFNα therapeutic response in patients with hepatocellular carcinoma [15]. Here, we used IFNα and an artificial analogue of U25 small nucleolar RNA (snoRNA) to modulate expression of innate immunity genes in breast cells and determined that activation of *IFIT3* expression is inversely correlated with its mRNA baseline level in primary breast cells and in immortalized breast cell lines.

## Methods

### Chemicals and antibodies

Cisplatin, doxorubicin, anastrozole, exemestane, and everolimus (afinitor) were purchased from Sigma-Aldrich (St. Louis, MO, USA). Phycoerythrin (PE)-conjugated mouse anti-human CD44 monoclonal (#MHCD4404) and fluorescein isothiocyanate (FITC)-conjugated mouse anti-human CD24 monoclonal (#MHCD4201) antibodies were purchased from Molecular Probes (Invitrogen, Carlsbad, CA, USA). FITC-conjugated mouse anti-human HER2 monoclonal and allophycocyanin (APC)-conjugated mouse anti human HER3 monoclonal (#2223535) antibodies were purchased from Sony Biotechnology Inc. (San Jose, CA, USA). APC- and FITC-conjugated IgG controls were from BD Biosciences.

### Cell cultures

MCF7, MDA-MB-231, and MCF10A cells were obtained from the Russian cell culture collection (Russian Branch of the ETCS, St. Petersburg, Russia). MDA-MB-231 cells were grown in Leibovitz media (L15, Sigma-Aldrich) supplemented with 10% fetal bovine serum (FBS; Gibco BRL Co., Gaithersburg, MD, USA), 2 mM L-glutamine, 250 mg/mL amphotericin B, and 100 U/mL penicillin/streptomycin. MCF7 cells were cultivated in Iscove’s modified Dulbecco’s media (IMDM; Sigma-Aldrich) with 10% FBS (Gibco BRL Co., Gaithersburg, MD, USA), 2 mM L-glutamine (Sigma-Aldrich), 250 mg/mL amphotericin B, and 100 U/mL penicillin/streptomycin (Gibco BRL Co., Gaithersburg, MD, USA). MCF10A were cultured in HuMEC Basal Serum-free medium (Gibco BRL Co., Gaithersburg, MD, USA) supplemented with HuMEC Supplement Kit (Gibco BRL Co., Gaithersburg, MD, USA).

### Human tissue specimens

Normal human breast tissue was obtained during size-reduction plastic surgery from healthy women at the Centre of New Medical Technologies (Novosibirsk, Russian Federation). Cancer tissue samples were obtained with informed consent from patients at the Novosibirsk Region Oncologic Dispensary (Novosibirsk, Russian Federation). The final diagnosis of cancer was confirmed by haematoxylin-eosin staining of paraffin blocks after the operation. One of the patients received six courses of chemotherapy with doxorubicin/cyclophosphamide before surgery. All patients gave written informed consent. The study protocol was approved by the Institute of Molecular Biology and Biophysics SB RAS Ethics Committee (Report#1 from March, 14 2017) in accordance with the Declaration of Helsinki of 1975. The fresh tumour and normal tissue specimens were immediately transferred into ice-cold DMEM medium (Gibco BRL Co., Invitrogen) supplemented with 100 U/mL penicillin, 100 μg/mL streptomycin, and 250 mg/mL amphotericin B.

### Primary cell culture preparation

Tissue specimens were mechanically dissociated using a scalpel and transferred to a solution of 20 mg/mL collagenase I (Gibco BRL Co., Invitrogen) in DMEM medium and incubated at 37 °C for 15 h on a shaking incubator (Grant Bio, Keison Products, UK). Specimens dissociated into single cells were washed with 10× excess of phosphate-buffered saline (PBS) and separated cells were collected by centrifugation at 300×*g*. Cells were plated in IMDM with 10% FBS and, after cell adhesion, 10 μM Rho-associated protein kinase (ROCK) inhibitor was added to the culture medium for 1 h and the medium in the plates was replaced with fresh complete IMDM medium. At the next passages, cells were cultured in complete IMDM medium supplemented with epithelial cell growth supplement (#6622, Cell Biologics, Chicago, IL, USA), Mito + Serum Extender (BD Biosciences - Discovery Labware, San Jose, CA, USA), 2 mM L-glutamine, 100 U/mL penicillin, 100 μg/mL streptomycin, and 250 mg/mL amphotericin B and were cultivated in 6-well plates at 37 °C in a humidified atmosphere containing 5% CO_2_. When 70–80% confluence was reached, cells were harvested using 0.05% trypsin/ethylenediaminetetraacetic acid (Sigma-Aldrich) and sub-cultured for further experiments.

### Cytotoxicity assay

Cell proliferation and survival were analysed using the iCELLigence RTCA (Real Time Cell Analyser) system by measuring cell-to-electrode responses of the cells seeded in 8-well E-plates with the integrated microelectronic sensor arrays (ACEA Biosciences Inc., San Diego, CA, USA). The functional unit of a cellular impedance assay is a set of gold microelectrodes fused to the bottom surface of plate well. The assay was performed as reported previously [16]. Briefly, cells were seeded in 8-well electronic plates at a density of about 1500 cells per well in a total volume of 200 μL of IMDM and were monitored in real-time mode with the iCELLigence RTCA system. After the initial 24 h of growth, the culture medium was replaced with fresh medium with dissolved drugs and monitored real-time. Cell index was calculated for each E-plate well by RTCA Software 1.2 (Roche Diagnostics, Meylan, France). Cell index is a parameter reflecting the impedance of electron flow caused by adherent cells. When cells reach the confluence, the CI value reaches the plateaus.

### Flow cytometry

Cells growing in 6-well plates were collected, fixed in 10% neutral buffered formalin, and incubated with labelled mouse anti-human antibodies (CD24 and CD44 or HER2/CD340 and HER3) for 30 min on ice in PBS supplemented with 10% normal goat serum. All analyses were performed using a FACSCantoII flow cytometer (BD Biosciences, Franklin Lakes, NJ, USA), and the data were analysed by FACSDiva Software (BD Biosciences). Cells were initially gated based on forward versus side scatter to exclude small debris, and ten thousand events from this population were collected. Control cells were treated with appropriate isotype FITC and APC-conjugated IgG (BD Biosciences).

### RNA transfection, INFα exposure, and total RNA isolation

Cells were seeded at 5 × 10^4^ cells/well in a 24-well plate and incubated for 24 h in a humidified incubator at 37 °C with 5% CO_2_. Analogues of snoRNAs were preincubated with Lipofectamine 3000 (Invitrogen) according to the manufacturer’s protocol and added to the culture medium at a final concentration of 50 nM. INFα was added to the culture medium at a final concentration of 40 IU/mL. After incubation for 21 h, total RNA was isolated by phenol-chloroform extraction using the Lira reagent (Biolabmix Ltd., Novosibirsk, Russia) according to the manufacturer’s protocol. The quality of total RNA was assessed by agarose gel electrophoresis or capillary electrophoresis with an Agilent 2100 Bioanalyzer, using 28S/18S > 2 or RIN > 8.0 criterion.

### Quantitative reverse transcription polymerase chain reaction (RT-PCR)

RT-PCR was performed in the one-tube reaction mixture BioMaster RT-PCR SYBR Blue (Biolabmix Ltd., Novosibirsk, Russia, www.biolabmix.ru) with gene-specific primers:

ERα: 5’-ATGATGAAAGGTGGGATACGA-3′ and 5’-CTGTTCTTCTTAGAGCGTTTGATC-3′; ERβ: 5′-TTGGATGGAGGTGTTAATGATG -3′ and 5’-GAAGTAGTTGCCAGGAGCATGT-3′; PGR: 5’-TCATTCTATTCATTATGCCTTACCA-3′ and 5’-GACTTCGTAGCCCTTCCAAAG-3′;Cyp19: 5’-TGCGAGTCTGGATCTCTGGA-3′ and 5’-GGGCCTGACAGAGCTTTCATA-3′; hypoxanthine-guanine phosphoribosyltransferase (HPRT): 5’-CATCAAAGCACTGAATAGAAAT-3′ and 5’-TATCTTCCACAATCAAGACATT-3′; glyceraldehyde 6-phosphate dehydrogenase (GAPDH): 5’-GAAGATGGTGATGGGATTTC-3′ and 5’-GAAGGTGAAGGTCGGAGT-3′; U6: 5’-GTGCTCGCTTCGGCAGCAC-3′ and 5’-GGGCCATGCTAATCTTCTC-3′; and *IFIT3*: 5’-GGCAGACAGGAAGACTTCTG-3′ and 5’-TTTCTGCTTGGTCAGCATGT-3′.

To compare PCR product yields, we performed real-time RT-PCR on a Bio-Rad iQ5 Cycler (Hercules, CA, USA) and Light Cycler 96 (Roche, Roche Diagnostics International, Switzerland) and used common models and algorithms for analysis of real-time PCR data realized in corresponding equipment software [17–19]. The quality of reference genes was assessed using geNorm (Qbase+). Mean values (± standard deviation) from three independent experiments are shown.

### Statistical analysis

Significance was determined using a two-tailed, Student’s *t*-test using OriginPro 2015 software. All error bars represent standard error of the mean.

## Results

**Fig. 1:**
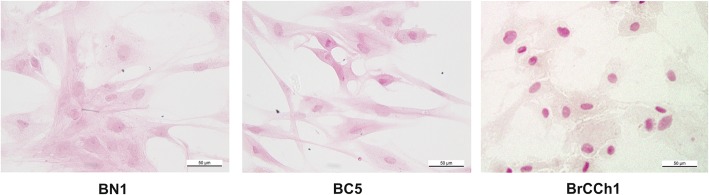
Representative images of BN1, BC5, and BrCCh1 cells (haematoxylin and eosin stain)

Cell cultures were established from the malignant breast tissue obtained after surgery by enzymatic disaggregation as described in the Methods. Carcinoma cells were prepared from the primary tumour (BC5) and the recurrent tumour after six courses of doxorubicin/cyclophosphamide chemotherapy (BrCCh1) while normal cells (BN1) were prepared from non-transformed breast tissue of healthy women. Morphological characteristics of cells grown for 1 week are presented in Fig. 1.

At this phase of culture, we observed a morphological heterogeneity of cells, such as long, flattened mesenchymal-like cells and epithelioid cells with the presence of the occasional multinucleated cell.

**Fig. 2:**
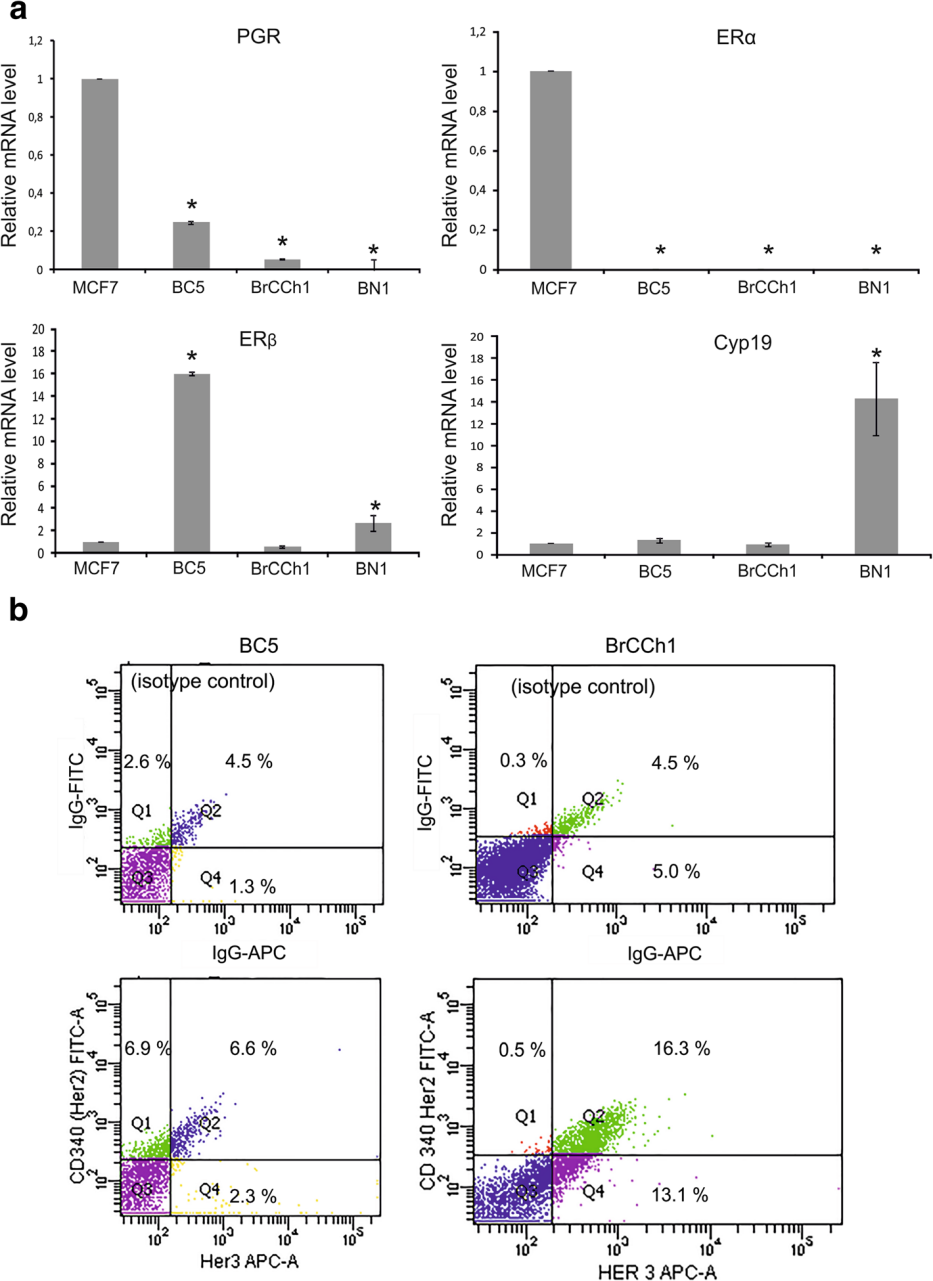
Expression of biological markers in MCF-7, BC5, BrCCh1, and BN1 cells. **a** Expression levels of the progesterone receptor (PGR), oestrogen receptor (ER) α and β, and Cyp19 mRNA in primary cell lines according to real-time polymerase chain reaction (PCR) analysis. The expression of specific mRNAs was normalised to the expression level of glyceraldehyde 6-phosphate dehydrogenase (GAPDH) mRNA. The expression levels of PGR, ERα, ERβ, and Cyp19 mRNA in the different cell lines are shown relative to their expression level in MCF-7 cells where it was set equal to 1. Statistical analysis included the results of two independent experiments (mean ± SD). * The difference between the experimental group and the control (MCF-7) group was statistically significant at *p* < 0.05. **b** Representative flow cytometry data of human epidermal growth factor receptor HER2(CD340)/HER3 positive cells in BC5 and BrCCh1 cells. Stained cells were gated according to isotype control samples so that these cells were negative to both HER2/HER3 (left bottom quadrant). Cells from right upper quadrant were accounted as HER2/HER3 double-positive cells

Primary cells were analysed for expression of PGR, ER, HER2, and HER3. To assess whether the established cell cultures express ERs, PGRs, and Cyp19, we analysed their specific mRNA compared with mRNA levels in the ER-positive human breast adenocarcinoma cell line MCF-7. mRNA signals from primary cells were defined as low/high if they were three times or more lower/higher than in MCF-7 cells, respectively. We revealed that BC5 carcinoma cells were PGR^low^/ERβ^high^/ERα^−^/Cyp19^+^, BrCCh1 cells that originated from the recurrent tumour were PGR^−^/ERβ^+^/ERα^−^/Cyp19^+^, and normal BN1 cells were PGR^−^/ERβ^+^/ERα^−^/Cyp19^high^ (Fig. 2a).

Expression of ERβ mRNA was 16-times higher in BC5 cells than MCF-7 cells. The expression of aromatase mRNA in BC5 and BrCCh1 cells was similar and match with aromatase mRNA expression in MCF-7 cells while BN1 cells expressed Cyp19A1 mRNA at a high level. ERα mRNA was not detected in all analysed cell cultures that is characterized these cells as ERα-negative (Fig. 2a).

**Fig. 3:**
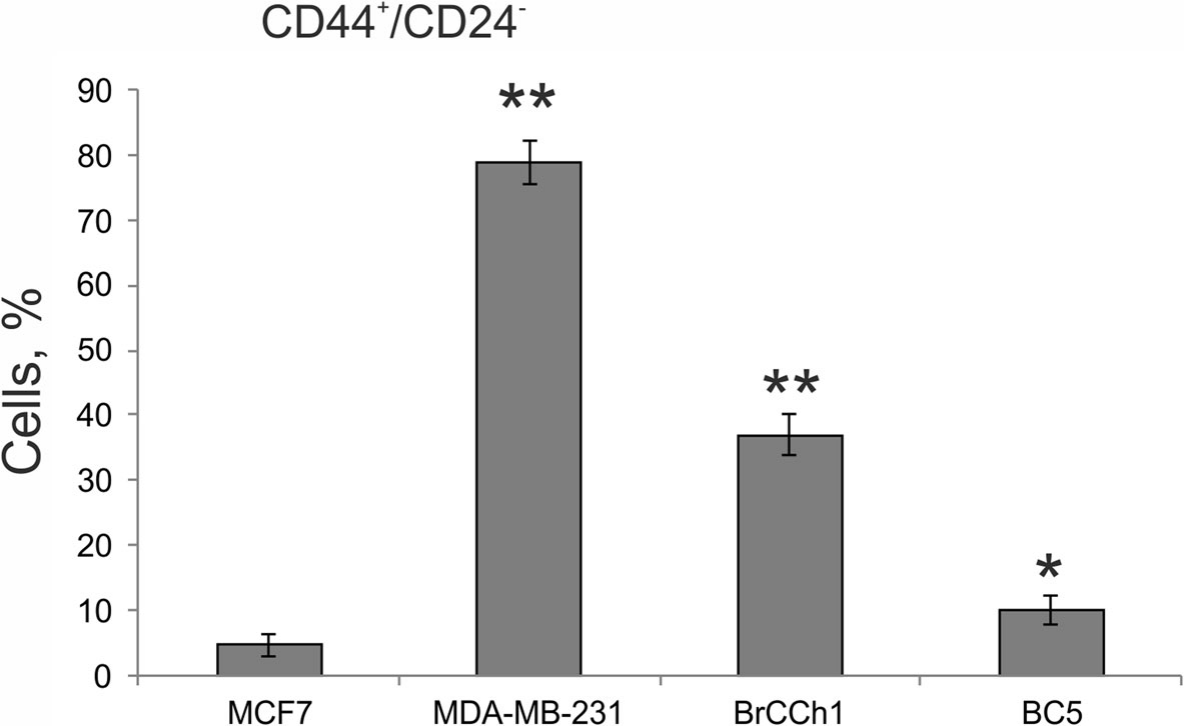
Relative contribution of the cancer stem cell-like CD44^+^/CD24^−^ subpopulations in cultures of breast cancer cells. Bar graph showing the percentage of CD44^+^/CD24^−^ cells detected by flow cytometry. Statistical analysis included the results of three independent experiments (mean ± SD). The difference between the experimental group and the MCF-7 group was statistically significant at p < 0.05 (*) and at *p* < 0.01 (**)

Overexpression of the tyrosine kinases HER2 (CD340) and HER3 is associated with decreased overall survival in breast cancer. Thus, these receptors play an important role in breast cancer. The HER2/HER3 heterodimer is a critical oncogenic unit associated with reduced relapse-free and decreased overall survival [20]. Here, HER2 and HER3 surface expression in BC5 and BrCCh1 cells was analysed by flow cytometry. Positivity for HER2 was defined as more than 10% cells stained for HER2, according to ASCO guideline, and for HER3 the cutoff value was the same [21]. We found that only BrCCh1 cells contain an essential HER3^+^ population (≈ 20%) and a HER2^+^/HER3^+^ double positive population (Fig. 2b). BrCCh1 cells containing about 10% HER2-positive population were defined low-positive for HER2. Analysis of a CSC-like population in BC5, BrCCh1, and BN1 cell lines was performed using a CD44/CD24 flow cytometry assay. We determined that approximately 37% of BrCCh1 cells exhibited a CD44^+^/CD24^−^ phenotype, which is associated with a poor outcome in patients [22], but the percentage of CD44^+^/CD24^−^ cells in BrCCh1 cells was lower than in MDA-MB-231 cells (Fig. 3).

**Fig. 4:**
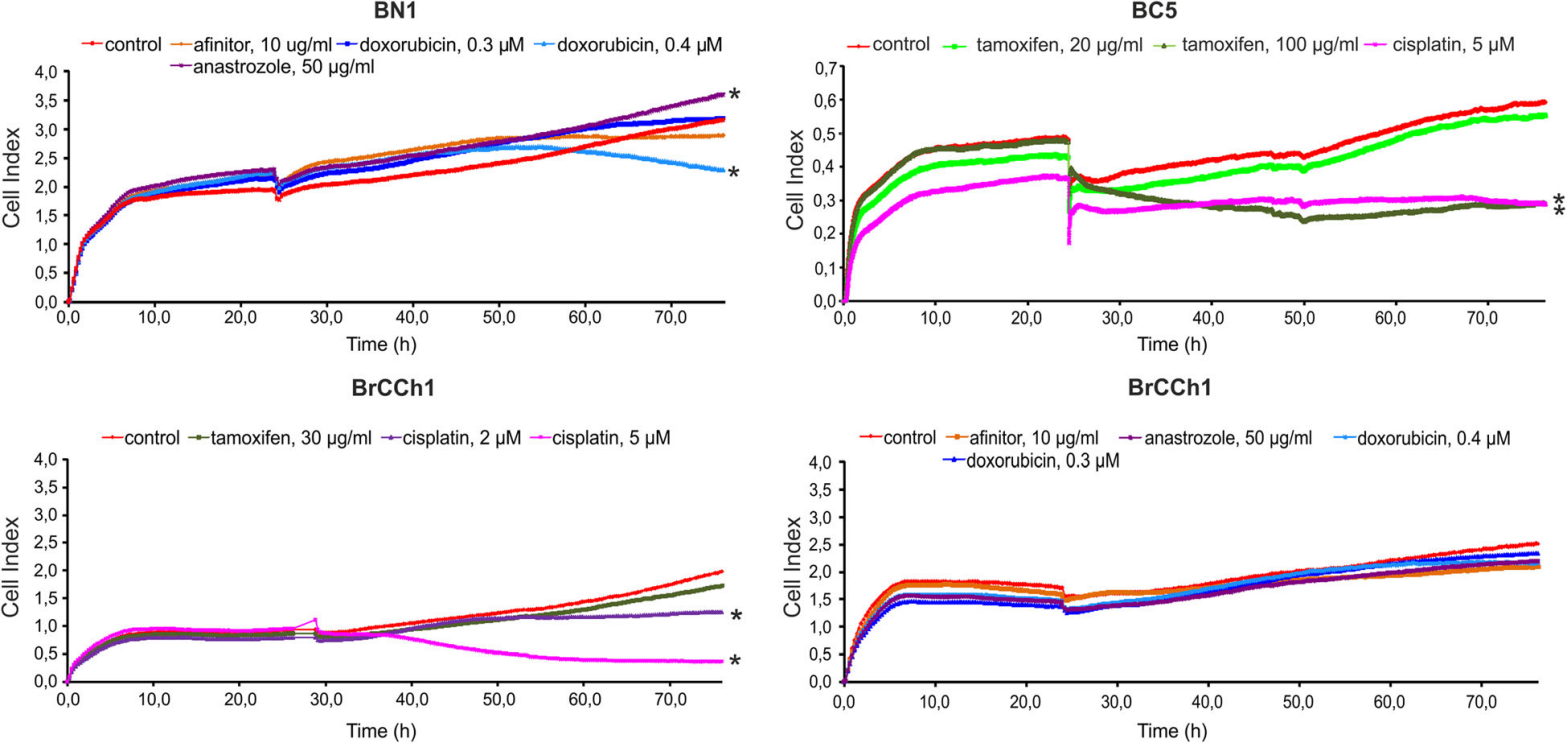
Influence of chemotherapeutic agents on the proliferation and death of BN1, BC5, and BrCCh1 breast cells. Cells were seeded into iCelligence 8-well plates and after 24 h were treated with substances listed in the legend. Curves represent the real-time monitoring of drug-mediated changes in the cell population (one of the three independent experiments). The difference between the experimental group and the control group (cells treated with PBS) was statistically significant at *p* < 0.05 (*)

Next, we estimated the sensitivity of BC5, BrCCh1, and BN1 breast cells to various chemotherapeutic agents: doxorubicin, cisplatin, tamoxifen, anastrozole, and the mTOR-inhibitor afinitor (everolimus), which is used for treatment of advanced hormone receptor-positive, HER2-negative breast cancer [23]. It was determined that nonmalignant BN1 cells were sensitive only to a high dose of doxorubicin (Fig. 4).

Anastrozole is an aromatase inhibitor that actively inhibits oestrogen conversion [24]. Both BN1 normal and BrCCh1 cancer cells were resistant to low concentrations (50 mkg/mL) of anastrozole. BC5 cells were sensitive to high doses of tamoxifen (100 mkg/mL) and less sensitive to 5 mkM of cisplatin than BrCCh1 cells (Fig. 4). BrCCh1 cells were sensitive only to 2 mkM of cisplatin, which allowed us to describe these cells as drug-resistant.

Previously, we analysed the influence of artificial small non-coding RNAs on gene expression in cancer cells and determined that transfection of MCF-7 breast cancer cells with artificial analogues of box C/D snoRNAs strongly induced activation of innate immunity genes, including *IFIT3* [14]. It is known that the induction of IFN/STAT1-related gene expression, which includes *IFIT3*, could be an early predictive marker of tumour response to chemotherapy in ER^−^ breast cancers [25].

To characterise the sensitivity of BC5, BrCCh1, and BN1 breast cells to the influence of innate immunity activators we used an artificial analogue of U25 box C/D snoRNA, and IFNα.

**Fig. 5:**
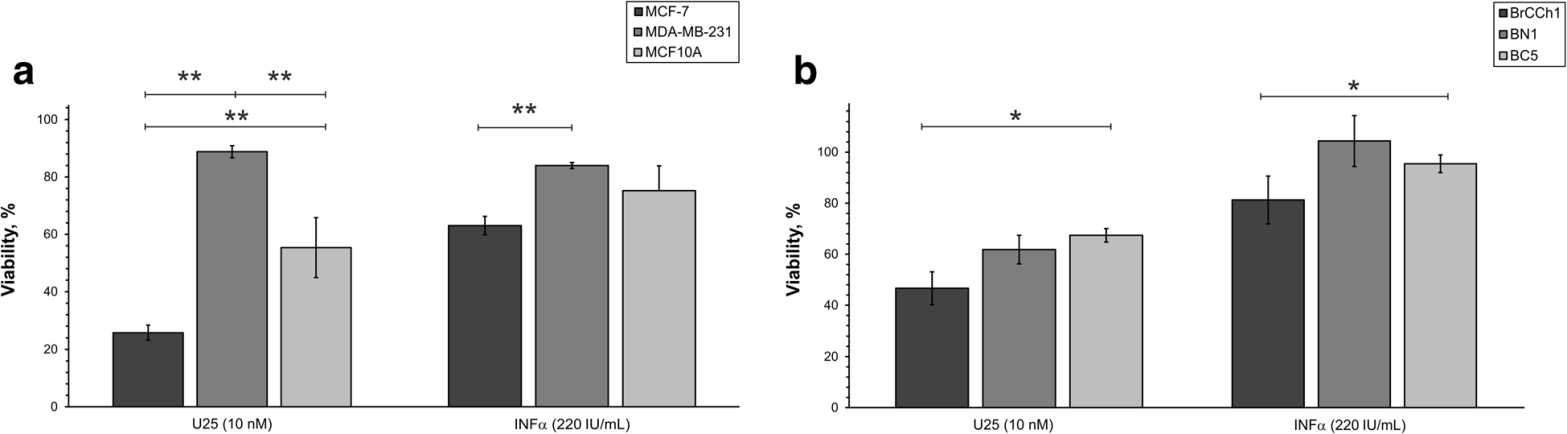
Cytotoxic activity of U25 and IFNα. Cells growing into iCelligence 8-well plates were treated with U25 (10 nM) or IFNα (220 U/mL) in the presence of Lipofectamine 3000. After 48 h of incubation the viability of treated cells was compared with that of control (treated with Lipofectamine 3000) cells (100%). **a** Percentage of viable cells in immortalized cell lines. **b** Percentage of viable cells in primary cultures. Statistical analysis included the results of three independent experiments (mean ± SD). The difference between the groups was statistically significant at *p* < 0.05 (*) and at *p* < 0.01 (**)

First, we analysed differences in sensitivity of primary and immortalized cells to the cytotoxic action of U25 snoRNA analogue and IFNα. We estimated effective concentrations of the substances that provide the optimal level of changes in cells viability using iCelligence RTCA assay. It was determined that U25 snoRNA analogue and IFNα induced decrease in cell viability in the range of ~ 5 to 70% at concentrations 10 nM and 220 UI/mL for U25 snoRNA analogue and IFNα, respectively (Fig. 5). The MCF-7 cells were the most responsive to the cytotoxic action of IFNα in the row MCF-7 > MCF10A > MDA-MB-231 (with viability of 63, 75 and 84%, correspondently Fig. 5a). BC5, BrCCh1, and BN1 breast cells did not show statistically significant differences in the sensitivity to snoRNA analogue and IFNα (Fig. 5b).

Taking into account that the interferon-α has the pleiotropic biologic activities, i.e. does not necessarily imply the proliferative or apoptotic cellular response, we analysed the activation of innate immunity processes in BC5, BrCCh1, and BN1 cells and in the immortalized breast cells MCF-7, MDA-MB-231, and MCF10A. For this we incubated cells with an artificial analogue of U25 box C/D snoRNA or IFNα and estimated the level of *IFIT3* mRNA using qRT-PCR.

**Fig. 6:**
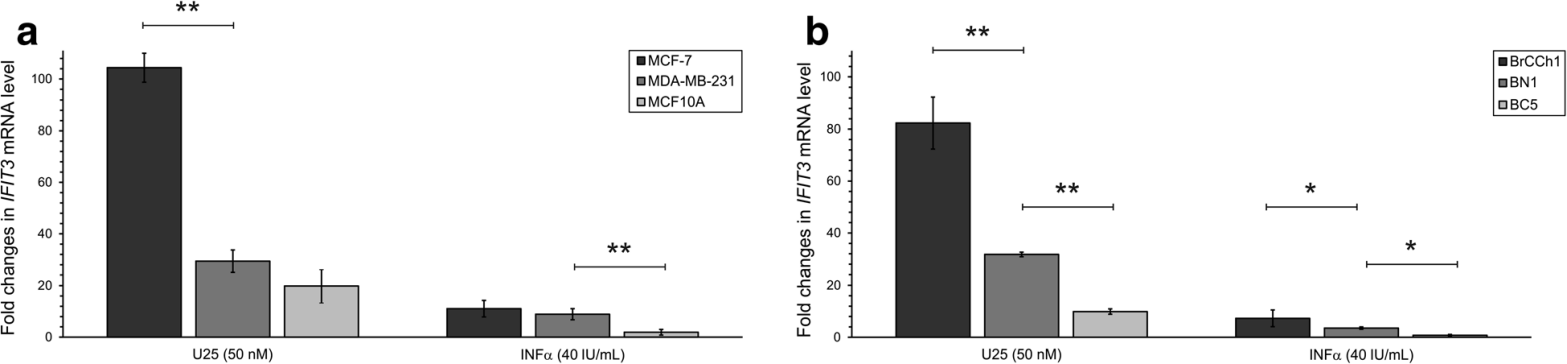
Expression of IFIT3 gene in human breast cell lines: **a** MCF7, MDA-MB-231, MCF10A, and primary cell lines **b** BrCCh1, BN1, BC5 after 24 h transfection with an analog of U25 C/D box in the presence Lipofectamine 3000. Quantitative RT-PCR values were normalized to level of *GAPDH*, *HPRT* and *RNU6* RNAs. Results are plotted relative to cells incubated with Lipofectamine 3000 for sample transfected with RNA and to non-treated cells for samples under INFα exposure. Data shown represent the mean ± SD from two independent experiments. The difference between the groups was statistically significant at *p* < 0.05 (*) and at *p* < 0.01 (**)

We determined that MCF7 cells were the most sensitive to artificial snoRNA, evidenced by the 100-fold increase in *IFIT3* mRNA (Fig. 6a). *IFIT3* mRNA was increased 30- and 20-fold following treatment with the immune-stimulating RNA (isRNA) in MDA-MB-231 cancer cells and MCF10A normal cells, respectively (Fig. 6a). It should be noted that the degree of changes in the *IFIT3* mRNA level caused by artificial snoRNA decreased in the following order: MCF-7 > MDA-MB-231 > MCF10A. Treatment with IFNα revealed a similar sensitivity: 11-, 9-, and 2-fold changes in *IFIT3* mRNA in MCF-7, MDA-MB-231, and MCF-10a cells, respectively. The increase in *IFIT3* mRNA induced by IFNα was lower than that for snoRNA (Fig. 6a and b).

**Fig. 7:**
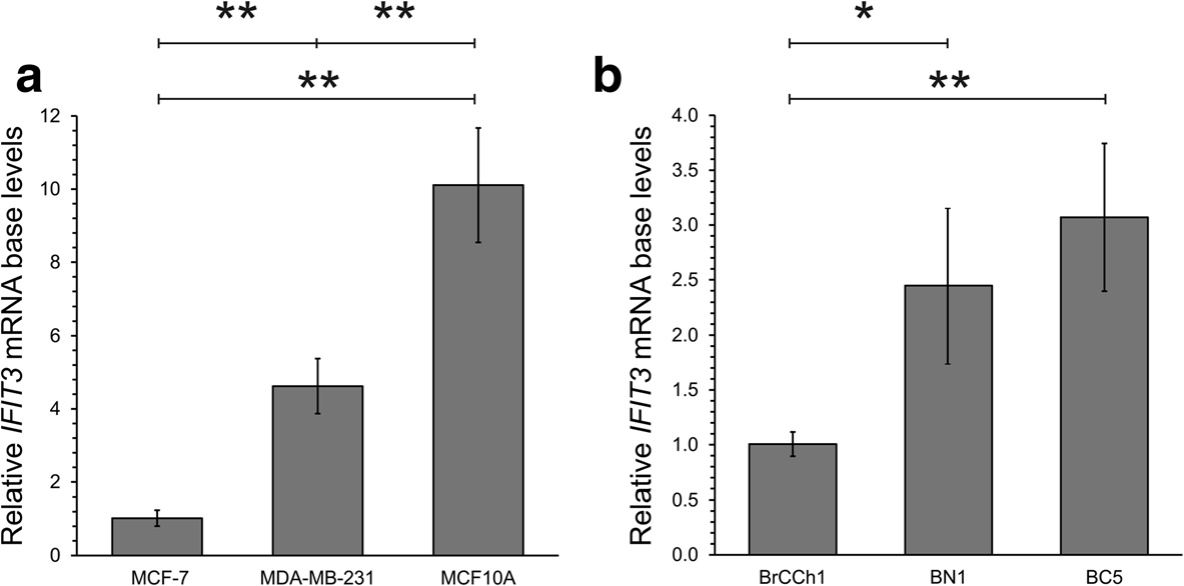
Baseline IFIT3 mRNA level in human breast cell lines: **a** MCF7, MDA-MB-231, MCF10A, and primary cell lines **b** BrCCh1, BN1, BC5. Quantitative RT-PCR values were normalized to level of *GAPDH*, *HPRT* and *RNU6* RNAs. **a** Results for MDA-MB-231 and MCF10A are presented as relative to level in MCF-7 cells; **b** results for BN1 and BC5 are presented as relative to level in BrCCh1 cells. Data shown represent the mean ± SD from two independent experiments. The difference between the groups was statistically significant at *p* < 0.05 (*) and at *p* < 0.01 (**)

To explain the differences in the activation of IFNα-inducible genes, we estimated the baseline level of *IFIT3* mRNA and determined that the activation of expression was inversely associated with the baseline level of *IFIT3* mRNA (Fig. 7). This suggests that the relative baseline level of *IFIT3* mRNA allows prediction of the sensitivity of cancer cells to the influence of innate immune modulators.

To confirm our hypothesis, we compared the mRNA levels of *IFIT3* in non-treated BC5, BrCCh1, and BN1 cells, as well as in the primary breast cells following exposure to artificial U25 box C/D snoRNA or IFNα. The highest transcriptional activation of *IFIT3* was found in BrCCh1 cells (82-fold for the artificial snoRNA and 7-fold for IFNα; Fig. 6b and Fig. 7b), while these cells are characterised with the lowest baseline level of *IFIT3* mRNA. BC5 cancer cells and normal BN1 cells demonstrated lower transcriptional activation that was inversely associated with the baseline expression level of the gene namely the snoRNA analogue induced a 10- and 32-fold increase in *IFIT3* mRNA, respectively. IFNα exposure led to 3.5-fold increase in *IFIT3* mRNA in BN1 normal cells and no significant change in BC5 cancer cells (Fig. 6 and Fig. 7). The Spearman’s rank correlation coefficient between the baseline level and the relative activation of *IFIT3* mRNA expression for all cell lines used was estimated at *r* = − 0.94 for the U25 snoRNA analogue as for IFNα (*p* < 0.05). Overall, for the immortalized and primary breast cells tested, we confirmed that the lower the *IFIT3* mRNA baseline level, the higher the degree of transcriptional activation under the influence of innate immunity activators.

## Discussion

The present study focused on the establishment and characterisation of primary breast cancer and nonmalignant cell lines and their application as a model for comparative analyses of sensitivity to therapeutic treatments. It is known that for breast cancers the initial investigation of molecular markers, which are important for the choice of therapy, includes analysis of the expression of steroid hormone receptors as well as determining HER2 status [26].

Adjuvant hormone therapy is effective following surgery among patients with ER-positive and/or PGR-positive breast cancers. Meanwhile, women with ER^−^/PGR^+^ or ER-poor/PGR^−^ tumours also receive adjuvant anti-oestrogen hormone therapy with hope that this treatment will benefit this group of patients [27, 28]. In the case of IHC assays of ER and PGR cut point for “positive” is 1% of stained cells, 1–10% ─ weakly positive and ≥ 10% ─ high positive [29]. RT-qPCR also can be used for the determination of breast cancer molecular subtype by quantification of ER and PGR mRNA levels [30].

In the present study, we compared the sensitivity of established hormone-positive BC5 cells and poor hormone-positive BrCCh1 cells to tamoxifen. As expected, according to ER/PGR status, only hormone-positive BC5 cells were sensitive to tamoxifen treatment (Fig. 4). Thus, we can conclude that PCR analysis of ER/PGR status in established cancer cell s predicted sensitivity to hormone therapy.

However, the clinical impact of CD24 and CD44 expression in tumours remains unclear, and further investigation will be necessary to evaluate the correlation between ER expression and CD44^+^/CD24^−^ cells in breast cancer. In general, CD44^+^/CD24^−^ cells appear most commonly in the TNBC subtype. Basal marker expression can complement with CD44^+^/CD24^−^ CSCs for an improved indicator for poor prognosis [28]. On the contrary, Horimoto and co-authors demonstrated that ER-positive patients with CD44^+^/CD24^−^ tumours had significantly longer disease-free survival than all other ER-positive patients [31]. Here, we observed high contribution of the CD44^+^/CD24^−^ population in the BrCCh1 ER-poor/PGR-poor/Her2^low^ tumour cell line, in which the phenotype is relatively close to TNBC (Fig. 3). Given the putative role of CSCs in tumour development and recurrence, we suppose that the low doxorubicin sensitivity of BrCCh1 cells that originated from the recurrent tumour is caused by CSC-like CD44^+^/CD24^−^ cells. BrCCh1 cells were also insensitive to tamoxifen, anastrozole, and afinitor, thus, it will be important to screen additional therapeutics to improve anticancer therapies for aggressive types of cancer. Epithelial-to-mesenchymal transition (EMT) has been described taking place in epithelial cells of breast cancers in vivo and in vitro [32]. EMT is visually defined by emergence of a fibroblastic-like cell morphology. Heterogeneous morphology of established BrCCh1 and BC5 cells, which were presented by flattened mesenchymal-like cells and epithelioid cells, can support thesis that during cultivation some of these cells undergo epithelial-to-mesenchymal transition. Such transition can be induced by CSCs, which have been detected in BrCCh1 and BC5 cells.

**Fig. 8:**
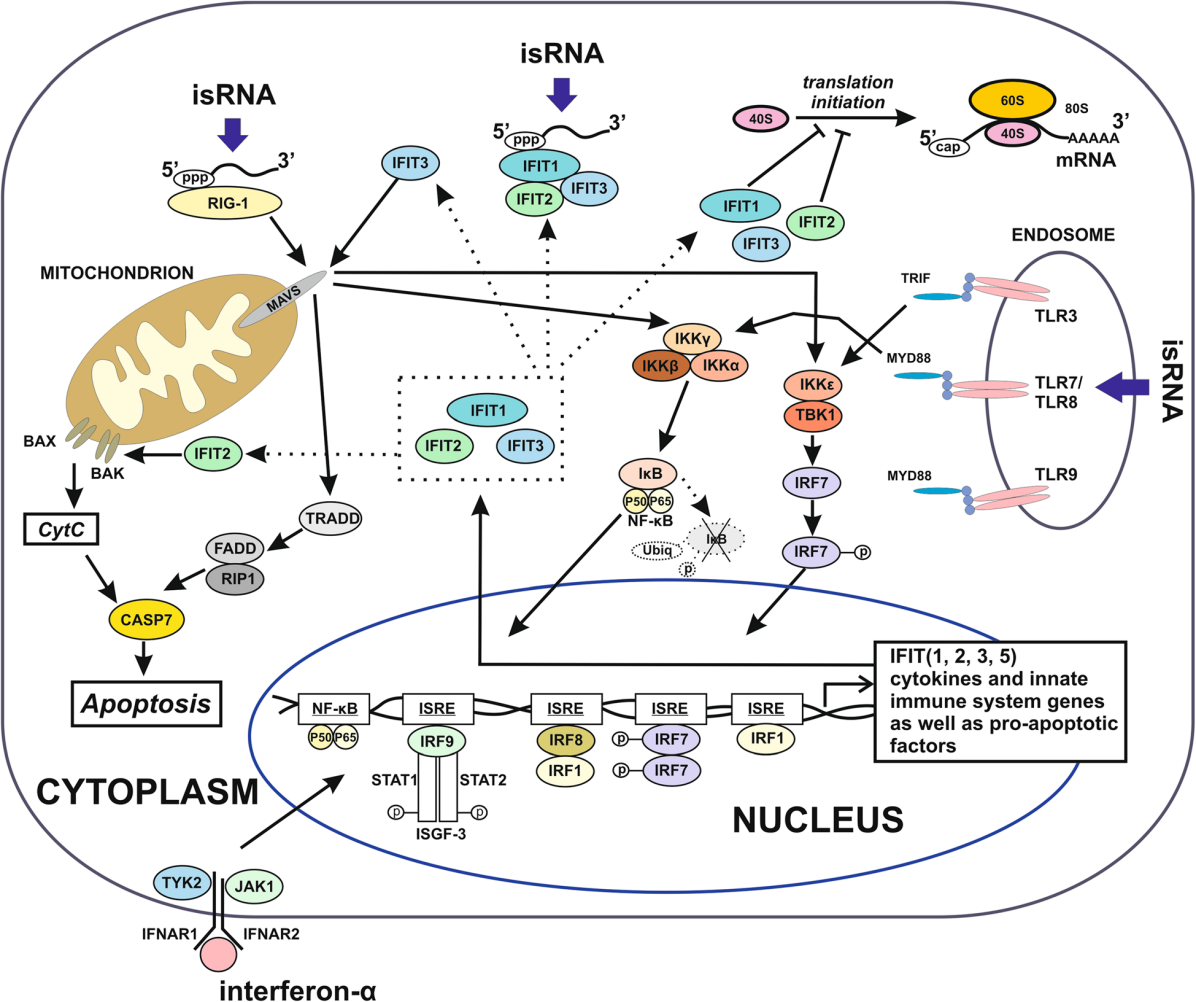
Relationship of immune-stimulating RNA (isRNA) actions, interferon-dependent pathways and IFIT functions in human cells (according to [35–37]). Solid arrows denote activation pathways of interferon-stimulated response elements (ISRE). Dashed arrows indicate known function IFIT family members

Activators of the immune system could become new powerful amplifiers of traditional chemotherapeutics for destroying cancers with poor prognosis [33]. On the other hand, artificial analogues of snoRNAs were recently shown to activate innate immunity in cancer cells [14, 34]. The action of isRNAs and interferons on cells can be described as results of interaction between ligands (RNAs or interferon) and receptors (Fig. 8). It is known, that interferon-α binds to the interferon-α/β receptor (IFNAR) and activates interferon-dependent cascades. Activation of the expression of interferon-sensitive genes in mammalian cells by RNA analogues is provided by a set of RNA-binding receptors, including TLRs, RIG-I, MDA-5, and PKR [35]. Earlier we found that artificial box C/D snoRNAs induced strong innate immune response. In particular, they upregulated transcription of the genes involved in cellular response to viral infection and foreign genetic material, such as RIG-I (DDX58), OAS1, MYD88, RNASEL, PKR (EIF2AK2), as well as interferon-dependent transcription factor STAT and IRF families (Fig. 8). Some products of interferon-activated genes are involved in cell death pathways and such activation can result in decrease of cell viability [14, 35–37].

In the present study, we analysed the effects of an artificial analogue of U25 snoRNA as well as INFα on *IFIT3* gene expression in primary breast cells and immortalized breast cell lines. We revealed that the activation of *IFIT3* expression was inversely correlated with its mRNA baseline level (Fig. 6 and Fig. 7).

As described by Zhao and co-authors, elevated expression of *IFIT3* enhanced anti- apoptotic activity and chemotherapy resistance in pancreatic ductal adenocarcinoma [38]. Our data demonstrate that a BrCCh1 chemoresistant cells with a large contribution of CSC-like population exhibits low baseline *IFIT3* mRNA with a prominent response to stimulation with snoRNA analogue or IFNα. On the contrary, nonmalignant breast MCF10A cells expressed relatively high *IFIT3* mRNA in the culture conditions, compared with MCF7 and MDA-MB-231 cells, and demonstrated modest response to xenogeneic RNA and INFα.

Yang studied the expression of *IFIT3* in hepatocellular carcinoma specimens following IFN therapy and showed that the activation of *IFIT3* expression correlates with patient survival [15]. In our study, comparing the data of *IFIT3* expression within particular cell lines following stimulation by immunomodulators and without stimulation we concluded that there was an inverse correlation in the intensity of the *IFIT3* response to immunostimulators and constitutive *IFIT3* mRNA expression. Elevated level of *IFIT3* in cancer cells can be regarded as alteration in activation pathways as well as known *IFIT3*-dependent downstream signalling (Fig. 8). It should be noted that the *IFIT3* level can be used as an indicator of cancer cells with null or low sensitivity to immune-stimulating agents. To establish molecular mechanisms determining these phenotypes of cancer cells the studies of mutations of *IFIT3* and its partner genes, copy number variations and transcription factor activity are needed.

## Conclusions

Here, we studied three primary cultures after propagation from human breast tissue and compared their response to conventional chemotherapeutics and to innate immunity stimulators with that of the immortalized breast cells MCF7, MDA-MB-231, and MCF10A. Our results suggest that when treated with xenogeneic RNA and INFα, the intensity of *IFIT3* mRNA expression is inversely associated with the baseline level of this mRNA in breast cancer cells. Summarizing our results and recently reported data, *IFIT3* expression can be considered a prognostic indicator of the efficacy of cancer immunotherapy. Because we did not observe any correlation of *IFIT3* expression and the ER/PGR/HER2 status of cancer cells, we suggest that *IFIT3* expression be tested regardless of ER/PGR/HER2 status if innate immunity activators will be used.

## Abbreviations

ASCO: American Society of Clinical Oncology
CSC: Cancer stem cells
ER: Estrogen receptors
HER: Human epidermal growth factor receptors
IFIT3: Interferon-induced protein with tetracopeptide repeat 3
INF: Interferons
isRNA: Immune-stimulating RNA
PGR: Progesterone receptors
TNBC: Triple negative breast cancer
